# A dynamical systems model of the effect of Locus Coeruleus firing on single trial cortical state dynamics

**DOI:** 10.1186/1471-2202-15-S1-P73

**Published:** 2014-07-21

**Authors:** Houman Safaai, Ricardo Neves, Oxana Eschenko, Nikos K Logothetis, Stefano Panzeri

**Affiliations:** 1 Center for Neuroscience and Cognitive Systems, Istituto Italiano di Tecnologia, Corso Bettini 31, 38068, Rovereto, Italy; 2 Department of Physiology of Cognitive Processes, Max Planck Institute for Biological Cybernetics, 72076 Tübingen, Germany

## 

Activity of sensory areas continuously varies reflecting both changes in external sensory stimuli and in internal states of the organism that do not necessarily always have a precise relationship to sensory inputs. An important aim in systems neuroscience is to develop quantitative models that may explain how state-dependent representations are generated and how they can be best interpreted. State-dependent modulations of cortical activity are in part mediated by changes in activity of various subcortical neuromodulatory nuclei. A prominent example is the Locus Coeruleus (LC), which can modulate both ongoing changes of cortical states and their responses to sensory inputs. However, a quantitative model of how the temporal fluctuations of LC firing affect ongoing and stimulus-driven primary cortical dynamics is currently missing.

Here we investigated how LC modulates the ongoing cortical states and the sensory information carried by cortical firing using a combination of neurophysiological experiments and data-driven dynamic-system models of cortical state changes. We performed simultaneous recordings of neural activity in primary somatosensory cortex and both ipsilateral and contralateral LC in urethane anaesthetized rats during spontaneous activity and during electrical stimulation of the contralateral hind paw. On the basis of this data, we have constructed a novel data-driven dynamical system model of cortical states dynamics. This model extends and generalizes recent simple effective models [1] by including the effect of firing of LC noradrenergic neurons.

We first fitted dynamic systems models of cortical states that either did or did not contain LC-cortical interactions. We found that models omitting the LC noradrenergic input to cortex tend to describe the dynamics of spontaneous activity of S1 cortex reasonably well. However, including ipsilateral (and to a lesser extent contralateral) LC activity as input to the models make the prediction of cortical states and of single trials responses much better. We then investigated which aspects of the LC dynamics help the model to increase cortical state predictability. We found that ipsilateral LC firing activity at low frequencies (< 10 Hz) correlated positively with slow (1-6 Hz) fluctuations of cortical power. The insertion of ipsilateral LC input to the model captured this dynamics by creating additional low frequency (1-6 Hz) state variation of model activity that correlated, both in power and phase, to those observed in real cortical activity. We finally investigated how these dynamical systems models can be used to predict single trial sensory evoked responses and to understand how this dynamics shapes the information representation of sensory stimuli. We derived an explicit mathematical rule predicting the trial-by-trial variability of cortical responses to stimuli arising from LC-modulated cortical state dynamics, and found that subtracting this variability from single trial cortical responses approximately doubles the amount of mutual information about the somatosensory stimuli that could be extracted from cortical responses.
